# Supplementary material for: Cellpose3: one-click image restoration for improved cellular segmentation
Source: Nat Methods. 2025 Feb 12;22(3):592–9. doi: 10.1038/s41592-025-02595-5 (PMC11903308; doi:10.1038/s41592-025-02595-5)
Supplement: Supplementary file 1 — Supplementary Table 1 [file 41592_2025_2595_MOESM1_ESM.pdf]

nature methods

Supplementary information https://doi.org/10.1038/s41592-025-02595-5

Cellpose3: one-click image restoration for improved cellular segmentation

In the format provided by the authors and unedited

model type	*Denoising*		*Deblurring*		*Upsampling*	
	cells	*nuclei*	*cells*	*nuclei*	*cells*	*nuclei*
degraded	0.429	0.393	0.391	0.402	0.425	0.492
per.+seg. loss	0.686	**0.708**	**0.638**	0.675	**0.665**	**0.652**
segmentation loss	**0.688**	0.704	0.637	**0.680**	0.658	0.644
reconstruction loss	0.634	0.697	0.584	0.642	0.604	0.586
Noise2Void	0.516	0.585	N/A	N/A	N/A	N/A
Noise2Self	0.522	0.499	N/A	N/A	N/A	N/A
retrain w/ noisy	0.631	0.618	N/A	N/A	N/A	N/A
one-click	0.663	0.699	0.625	0.639	0.640	0.604

Supplementary Table 1. Average precision at 0.5 IoU threshold for various denoising/deblurring/upsampling strategies.
