# Supplementary material for: Cellpose3: one-click image restoration for improved cellular segmentation
Source: Nat Methods. 2025 Feb 12;22(3):592–9. doi: 10.1038/s41592-025-02595-5 (PMC11903308; doi:10.1038/s41592-025-02595-5)
Supplement: Supplementary file 2 — Reporting Summary [file 41592_2025_2595_MOESM2_ESM.pdf]

nature portfolio

Corresponding author(s): Carsen Stringer

Last updated by author(s): Feb 29, 2024

Reporting Summary

Nature Portfolio wishes to improve the reproducibility of the work that we publish. This form provides structure for consistency and transparency in reporting. For further information on Nature Portfolio policies, see our Editorial Policies and the Editorial Policy Checklist.

Statistics

For all statistical analyses, confirm that the following items are present in the figure legend, table legend, main text, or Methods section.

n/a	Confirmed	
☐	☒	The exact sample size (*n*) for each experimental group/condition, given as a discrete number and unit of measurement
☐	☒	A statement on whether measurements were taken from distinct samples or whether the same sample was measured repeatedly
☒	☐	The statistical test(s) used AND whether they are one- or two-sided<br>*Only common tests should be described solely by name; describe more complex techniques in the Methods section.*
☒	☐	A description of all covariates tested
☒	☐	A description of any assumptions or corrections, such as tests of normality and adjustment for multiple comparisons
☒	☐	A full description of the statistical parameters including central tendency (e.g. means) or other basic estimates (e.g. regression coefficient) AND variation (e.g. standard deviation) or associated estimates of uncertainty (e.g. confidence intervals)
☒	☐	For null hypothesis testing, the test statistic (e.g. *F*, *t*, *r*) with confidence intervals, effect sizes, degrees of freedom and *P* value noted<br>*Give P values as exact values whenever suitable.*
☒	☐	For Bayesian analysis, information on the choice of priors and Markov chain Monte Carlo settings
☒	☐	For hierarchical and complex designs, identification of the appropriate level for tests and full reporting of outcomes
☒	☐	Estimates of effect sizes (e.g. Cohen's *d*, Pearson's *r*), indicating how they were calculated

Our web collection on statistics for biologists contains articles on many of the points above.

Software and code

Policy information about availability of computer code

Data collection	Scanimage software v2022.1.0 (open source) was used to collect calcium imaging data from awake mice using a two-photon mesoscope (Thorlabs 2PRAM microscope). PsychToolbox was used to present visual stimuli during the experiments.
Data analysis	cellpose==3.0, csbdeep==0.7.4, efficientnet-pytorch==0.7.1,fastremap==1.14.0, imagecodecs==2023.9.18, imageio==2.31.6, jupyter==1.0.0, keras==2.14.0, matplotlib==3.8.0, n2v==0.3.2, numba==0.58.0, numpy==1.25.2, nvidia-cuda-runtime-cu11==11.8.89, nvidia-cuda-runtime-cu12==12.1.105, opencv-python-headless==4.8.1.78, python==3.9.18, scipy==1.11.3, segmentation-models-pytorch==0.3.3, tensorflow==2.14.0, tifffile==2023.9.26, timm==0.9.2, torch==2.1.0, torchvision==0.16.0, noise2self github (latest release, 2019)

For manuscripts utilizing custom algorithms or software that are central to the research but not yet described in published literature, software must be made available to editors and reviewers. We strongly encourage code deposition in a community repository (e.g. GitHub). See the Nature Portfolio guidelines for submitting code & software for further information.

Data

Policy information about availability of data

All manuscripts must include a data availability statement. This statement should provide the following information, where applicable:

- Accession codes, unique identifiers, or web links for publicly available datasets
- A description of any restrictions on data availability
- For clinical datasets or third party data, please ensure that the statement adheres to our policy

We generated a new clean/noisy dataset using two-photon calcium imaging, available at https://doi.org/10.25378/janelia.27854442. The 'cyto2' dataset is publicly available at https://www.cellpose.org/dataset, and the other datasets were generated and shared by other labs.

Human research participants

Policy information about studies involving human research participants and Sex and Gender in Research.

Reporting on sex and gender	N/A
Population characteristics	N/A
Recruitment	N/A
Ethics oversight	N/A

Note that full information on the approval of the study protocol must also be provided in the manuscript.

Field-specific reporting

Please select the one below that is the best fit for your research. If you are not sure, read the appropriate sections before making your selection.

☒ Life sciences ☐ Behavioural & social sciences ☐ Ecological, evolutionary & environmental sciences

For a reference copy of the document with all sections, see nature.com/documents/nr-reporting-summary-flat.pdf

Life sciences study design

All studies must disclose on these points even when the disclosure is negative.

Sample size	The sample size in this study was nine large datasets of cellular and nuclear images (2D), which each consisted of several classes of images from different cell types, tissues or imaging modalities. This dataset size was determined by the availability of large-scale fully annotated datasets: it is a substantial effort to create these datasets. These datasets were sufficient for determining the performance of models because they spanned imaging modalities and cell types with various morphologies.
Data exclusions	We excluded 5 test images from DeepBacs which were labeled in a different image modality (defined in Methods).
Replication	All the models were trained on large datasets (~800 cyto images, or ~1000 nuclei images, or ~8000 images across all nine datasets), and the training of each model takes up to 24 hours on an A100 GPU, so we did not perform multiple training replications for most analyses. We think this is reasonable because the datasets are so large. All attempts at replication were successful (e.g. we ran multiple seeds for Figure S1 which attained similar performance).
Randomization	There was no splitting of samples or organisms in this study to perform comparisons of experimental groups.
Blinding	There was no splitting of samples or organisms in this study to perform comparisons of experimental groups, so blinding is not applicable to this study.

Reporting for specific materials, systems and methods

We require information from authors about some types of materials, experimental systems and methods used in many studies. Here, indicate whether each material, system or method listed is relevant to your study. If you are not sure if a list item applies to your research, read the appropriate section before selecting a response.

Materials & experimental systems

n/a	Involved in the study	
☒	☐	Antibodies
☒	☐	Eukaryotic cell lines
☒	☐	Palaeontology and archaeology
☐	☒	Animals and other organisms
☒	☐	Clinical data
☒	☐	Dual use research of concern

Methods

n/a	Involved in the study	
☒	☐	ChIP-seq
☒	☐	Flow cytometry
☒	☐	MRI-based neuroimaging

Animals and other research organisms

Policy information about studies involving animals; ARRIVE guidelines recommended for reporting animal research, and Sex and Gender in Research

Laboratory animals	riboL1-jGCaMP8s mouse, sex female, aged 4 months.
Wild animals	No wild animals used.
Reporting on sex	Sex-based analyses not required - neural activity not analyzed.
Field-collected samples	No field-collected sample.
Ethics oversight	IACUC board at HHMI Janelia Research Campus approved the research in the study.

Note that full information on the approval of the study protocol must also be provided in the manuscript.
